# Development and Characterization of New SSR Markers in *Sclerotinia sclerotiorum* Using Genomic and Variant Analysis

**DOI:** 10.3390/pathogens14070610

**Published:** 2025-06-20

**Authors:** Dong Jae Lee, Young-Joon Choi

**Affiliations:** 1 Department of Biological Science, Kunsan National University, Gunsan 54150, Republic of Korea; ing7629@gmail.com; 2 Center for Convergent Agrobioengineering, Kunsan National University, Gunsan 54150, Republic of Korea

**Keywords:** simple sequence repeat, polymorphic markers, population genetics, InDel variation

## Abstract

*Sclerotinia sclerotiorum* is a globally distributed fungal pathogen responsible for significant agricultural losses across a wide range of crops. This study aimed to develop polymorphic simple sequence repeat (SSR) markers by whole-genome resequencing of three Korean isolates and a public reference genome. A total of 16,885 SSR motifs were identified, of which 368 overlapped with polymorphic insertion–deletion (InDel) sites across the four genomes. From these, 12 SSR markers were selected based on polymorphism information content and amplification quality. Validation across the 28 isolates in Korea revealed high levels of genotypic diversity, suggesting that each isolate is a unique haplotype, although *S. sclerotiorum* is homothallic and clonally propagated. This multi-genome approach provides robust resources for genotyping, molecular diagnostics, and epidemiological surveillance of *S. sclerotiorum*.

## 1. Introduction

*Sclerotinia sclerotiorum* is a cosmopolitan necrotrophic fungus that affects more than 500 plant species, including economically important crops such as canola, lettuce, oilseed rape, soybean, and sunflower [1,2,3]. Its ability to produce long-lived sclerotia enables its prolonged survival in the soil and contributes to its persistence across diverse agroecosystems, thus making it a significant threat to global agriculture [4].

Previous population studies of *S. sclerotiorum* isolates have used various approaches, including mycelial compatibility assays, molecular genotyping, and aggressiveness testing for selected host cultivars [5,6,7,8,9]. Microsatellite and simple sequence repeat (SSR) markers have been used for genotyping *S. sclerotiorum* isolates [10]. SSR markers are widely recognized for their high polymorphism, codominant inheritance, and technical ease, making them particularly useful for population genetics, gene mapping, and diagnostic applications [11,12]. In *S. sclerotiorum*, SSRs have been used to study clonal diversity, host adaptation, and regional differentiation [13,14,15,16,17,18,19]. Despite its broad host range and global distribution, previous population studies have frequently reported the low overall genetic diversity of *S. sclerotiorum*, primarily because of its clonal reproductive strategy and limited recombination [20,21]. Although most investigations suggest that *S. sclerotiorum* predominantly reproduces asexually, occasional genetic recombination events, often interpreted as outcrossing, have also been reported [22,23,24]. These findings underscore the need for robust molecular tools to investigate population structure and evolutionary adaptation. In addition, previous SSR-based studies relied on a small number of loci derived from expressed sequence tags (ESTs) or partial genomic libraries, limiting their resolution and genome-wide applicability. With the advent of next-generation sequencing, whole-genome data now enable the systematic and comprehensive identification of polymorphic SSR loci across different genomes.

In the present study, we performed whole-genome resequencing of three *S. sclerotiorum* isolates, combined with insertion–deletion (InDel) variant analysis and SSR motif discovery, to construct a new set of polymorphic SSR markers. These markers aim to support high-resolution population studies, molecular diagnostics, and the monitoring of pathogen evolution and spread in agricultural systems.

## 2. Materials and Methods

### 2.1. Fungal Isolates and G-DNA Extraction

Twenty-eight isolates of *S. sclerotiorum* were obtained from the Korean Agricultural Culture Collection (KACC; Rural Development Administration, Jeonju, Republic of Korea) and are listed in Table 1. Each isolate was cultured on potato dextrose agar (PDA; Difco, Detroit, MI, USA) and incubated at 25 °C for five days in the dark. Genomic DNA was extracted from mycelia harvested from the isolates using a NucleoSpin Plant II Kit (Macherey-Nagel, Düren, Germany). DNA quality and quantity were evaluated using a Qubit 1X dsDNA High Sensitivity Kit with a Qubit Fluorometer (Thermo Fisher Scientific, Waltham, MA, USA).

### 2.2. Sequencing and Preprocessing

Whole-genome sequencing was performed on three *S. sclerotiorum* isolates (KACC 42223, 47260, and 410245) at Phyzen (Seongnam-si, Republic of Korea). Libraries were prepared using the TruSeq Nano DNA Kit (Illumina, San Diego, CA, USA), and paired-end sequencing (2 × 151 bp) was performed using the NovaSeq X platform. Adapter sequences and low-quality bases (Q < 20) were trimmed using Trimmomatic v0.39 [25]. Reads shorter than 50 bp were excluded from analysis. The overall pipeline for SSR marker development and analysis is illustrated in the Supplementary Data (Figure S1).

### 2.3. Read Alignment and Variant Calling

The high-quality reads were aligned to the *S. sclerotiorum* strain 1980 UF-70 reference genome (GCA_001857865.1) [26,27] using the Burrows–Wheeler Aligner (BWA-MEM) algorithm implemented in the Sentieon DNAseq pipeline v202112.01 (Sentieon, Inc., San Jose, CA, USA) [28]. The SAM files were converted to BAM and sorted, and PCR duplicates were marked. Variant calling was performed using the Sentieon Haplotyper, and the raw VCF files were filtered to retain biallelic variants with a genotype call rate of 1 and a read depth (DP) between 5× and 100×. SNP and InDel were annotated using SnpEff v5.0e [29]. False-positive SNPs were then removed from the raw variants using GATK v4.4.0.0 [30], and hard filtering was applied under the following conditions, GATK hard filtering was performed to remove low-quality variants using the following thresholds: depth (QD < 2.0), fisher strand (FS > 60.0), mapping quality (MQ < 40.0), mapping quality rank sum test (MQRankSum < −12.5), and read position rank sum test (ReadPosRankSum < −8.0), for SNP, while QD < 2.0, FS > 200.0, and ReadPosRankSum < −20.0 for InDel. Additional filtering, including allele depth (AD) and minor allele frequency (MAF), was performed using VCFtools v0.1.17 [31].

### 2.4. SSR Motif Discovery, Primer Design, and PCR Amplification

SSR motif discovery was first conducted by scanning the whole-genome sequences of the three Korean isolates and the reference genome using Phyzen (Seongnam-si, Republic of Korea) software. Repeat motifs ranging from 2 to 10 bp in length were identified, with minimum thresholds set at ≥5 for dinucleotides, ≥4 for trinucleotides, and ≥3 for all higher-order motifs. Polymorphic SSR candidates were defined as those exhibiting a repeat number difference of two or more between the four genomes. For each polymorphic SSR locus, primers were designed using Primer3 [32] with the following parameters: product size = 100–250 bp, primer size = 18–26 bp (optimum 22 bp), melting temperature = 55–62 °C (optimum 58 °C), and GC content ≤59%. Uniquely mapped primer pairs were selected for screening downstream polymorphisms. The PCR reaction mixture had a final volume of 25 μL, including AccuPower PCR Premix (Bioneer, Daejeon, Republic of Korea), 1 μL of G-DNA (1 ng/μL), 0.4 μM of each primer, and 0.8 μg/μL of bovine serum albumin (Biosesang, Seongnam, Republic of Korea), as well as nuclease-free water (Sigma-Aldrich; Merck, St. Louis, MO, USA). PCR amplification was performed with the following conditions: initial denaturation at 95 °C for 10 min, followed by 36 cycles of denaturation at 95 °C for 40 s, annealing at 50 °C for 40 s, elongation at 72 °C for 50 s, and a final elongation at 72 °C for 5 min. The PCR products of the SSR markers were subjected to electrophoresis and sequenced by Macrogen (Daejeon, Republic of Korea).

### 2.5. Detection of Polymorphic SSRs, Marker Selection, and Phylogenetic Analysis

Polymorphic SSR markers were identified by comparing SSR loci with InDel variants obtained from the whole-genome resequencing of the three *S. sclerotiorum* isolates against the reference genome. This comparative analysis revealed 368 SSR loci that overlapped with polymorphic InDel regions, indicating potential variations in the repeat numbers among the four genomes. To optimize the marker set for downstream applications, SSR loci were filtered using four criteria: (1) the presence of intergenomic repeat number differences of at least two, (2) even chromosomal distribution to ensure genome-wide representation, (3) the reliable performance of the designed primers under conventional PCR amplification, and (4) the presence of more than one distinct SSR motif pattern observed through the sequencing of PCR products. The candidate SSR markers were then validated across 28 *S. sclerotiorum* isolates by assessing amplification efficiency and sequencing quality. Finally, 12 high-quality SSR markers were retained for further genetic analysis.

### 2.6. Genetic and Phylogenetic Analyses

Genetic diversity among the 28 isolates was evaluated using the number of alleles (NA), expected heterozygosity (HE) [33], and polymorphism information content (PIC) [34] values. NA and HE were calculated using GenAlEx 6.51 b2 [35]. PIC values were estimated using Cervus v3.07 [36], following the classification of Botstein et al. [34], where PIC > 0.5 is considered highly informative, 0.25 < PIC ≤ 0.5 reasonably informative, and PIC < 0.25 slightly informative. Genetic relationships among the isolates were analyzed by computing the Euclidean distances based on SSR repeat number profiles. These distance values were used in order to construct an unweighted pair group method with an arithmetic mean (UPGMA) dendrogram using the genetic distance matrix generated using GenAlEx 6.51 b2. The resulting phylogenetic tree was visualized and edited using MEGA 12 [37].

## 3. Results

### 3.1. Preprocessing and Read Alignment

The whole-genome sequencing of the three *S. sclerotiorum* isolates (KACC42223, KACC47260, and KACC410245) generated between 18.0 and 22.4 million raw reads per sample (Table 2). After quality trimming, 84.6% to 94.1% of the reads were retained. These high-quality reads provided sufficient coverage for accurate variant calling, with average depths ranging from 48.99x to 71.14x across all three isolates. The alignment of the trimmed reads to the *S. sclerotiorum* reference genome (GCA_001857865.1) using BWA-MEM revealed that 74.58% to 85.67% of reads were successfully mapped, depending on the isolate. Genome-wide coverage at a minimum depth of 1× exceeded 97% for all samples, confirming uniform representation. However, coverage at higher thresholds (20× and 50×) varied among isolates, with KACC 47260 exhibiting the highest overall depth and KACC 410245 exhibiting the lowest overall depth. The genome sizes of the three *S. sclerotiorum* isolates were 38.18 Mb, 38.25 Mb, and 37.83 Mb, respectively.

### 3.2. Variant Detection and Filtering

Variant calling across the three isolates initially produced 183,126 raw variants. After stringent filtering to retain only the biallelic sites with complete genotype calls and an appropriate read depth (5×–100×), 76,603 high-confidence variants remained (Table 3). Among these, 88.8% were found to be SNPs and 11.2% were InDels. Functional annotation using SnpEff revealed that 67.3% of the variants were located in intergenic regions, and 32.7% were located within genic regions. Within the coding regions, non-synonymous (9526) and synonymous (9470) mutations were found to be most common, indicating their potential contributions to functional diversity.

### 3.3. SSR Motif Discovery and Marker Selection

**Figure 1:**
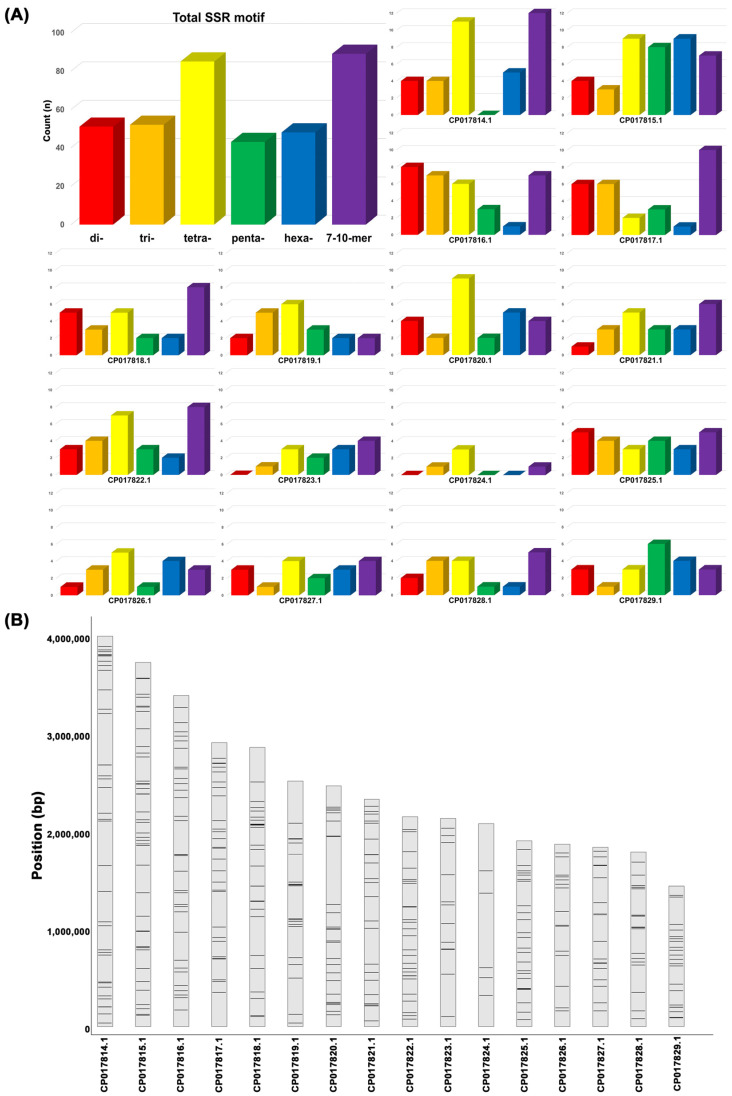
The genomic distribution and abundance of SSR motifs in *Sclerotinia sclerotiorum*. (**A**) The total counts of SSR motifs grouped by repeat unit length (2–10 bp), with tetranucleotide motifs showing the highest frequency. (**B**) The chromosomal locations of 368 polymorphic SSR variants mapped across the 16 scaffolds (CP017814.1—CP017829.1) of the reference genome (GenBank assembly accession: GCA_001857865.1). Regions of higher density indicate potential SSR clustering hotspots.

Comparative analysis of whole-genome resequencing data from the three *S. sclerotiorum* isolates and a reference genome identified 16,885 SSR motifs, comprising dinucleotide (2051), trinucleotide (2267), and tetranucleotide (6556) motifs. Among them, 368 SSR loci overlapped with polymorphic InDel regions and exhibited variations in repeat numbers across the genomes (Figure 1A). Among the selected motifs, tetranucleotide repeats (85) were the most abundant, while other repeat types, including di- (51), tri- (52), hexa- (48), and higher-order motifs (89), were more evenly distributed across the loci (Figure 1A). To ensure genomic representation, SSR loci were intentionally selected across different chromosomes, resulting in a balanced chromosomal distribution of the marker set (Figure 1B), with the number of polymorphic InDel-linked SSRs ranging from 5 (e.g., CP017824.1) to 40 (e.g., CP017815.1) (Figure S2). From this pool, 36 candidate SSR markers were further selected based on two criteria: the presence of repeat number differences ≥2 among the four genomes and chromosomal distribution balance. Of these, 12 SSR markers were selected based on successful PCR amplification, high-quality sequencing, and discriminatory power across a panel of 28 isolates.

### 3.4. SSR Polymorphism

**Figure 2:**
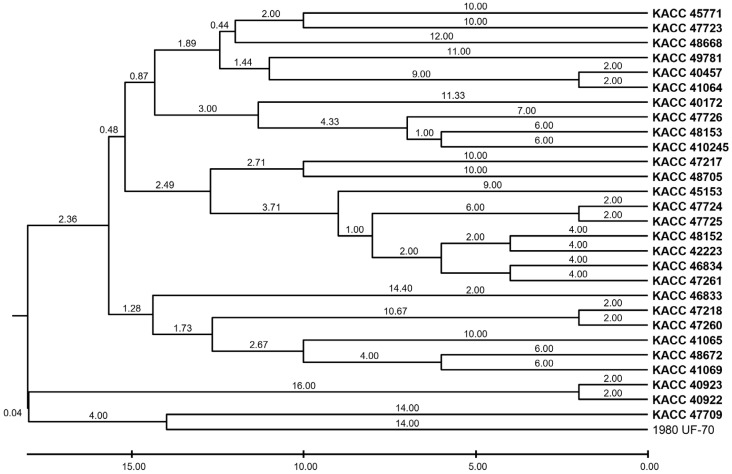
UPGMA dendrogram of *Sclerotinia sclerotiorum* isolates based on SSR repeat variation.

Twelve SSR markers were used to evaluate genetic variation using NA, HE, and PIC (Table 4). The PIC values of the markers ranged from 0.1862 (SS29–677) to 0.9014 (SS23–1015), with four markers—SS15–1627, SS16–359, SS17–256, and SS23–1015—exhibiting high informativeness (PIC > 0.80). The most polymorphic locus, SS23–1015, had 16 alleles, indicating high allelic richness. In contrast, SS16–70 and SS28–205 exhibited the lowest NA values (3 and 2, respectively) and relatively low PIC values, suggesting limited variation among the isolates at these loci. To explore the genetic relationships among the isolates, UPGMA clustering was performed based on SSR repeat number variations (Figure 2). The resulting dendrogram revealed distinct groupings that reflect the degree of population differentiation. However, these clusters showed no correlation with geographic origins, host associations, or clonal lineages within the *S. sclerotiorum* population studied.

## 4. Discussion

This study has used a genome-wide comparative approach to develop a set of polymorphic SSR markers in *S. sclerotiorum* by integrating repeat motif detection with InDel-based variation analysis of the whole-genome resequencing of three Korean isolates. This strategy represents a methodological advancement over previous SSR development studies that relied solely on a single reference genome (e.g., Sirjusingh and Kohn [10]), as it directly reflects inter-isolate genomic variation, enhancing marker informativeness while minimizing the inclusion of non-polymorphic loci.

The genome sizes of the three Korean isolates, viz., KACC42223, KACC47260, and KACC410245, ranged from 37.83 to 38.25 Mb, which are marginally smaller than those of previously sequenced strains such as 1980 UF-70 (38.9 Mb), ESR-01 (40.98 Mb), and WH6 (38.95 Mb) [26,27,38,39,40]. These differences suggest that the genome size of *S. sclerotiorum* may vary among isolates, which could be attributed to repetitive element content or structural variation.

The final set of 12 SSR markers, selected based on repeat number variation (≥2) and balanced chromosomal distribution, exhibited high amplification efficiency and polymorphisms among 28 Korean isolates. The allele count ranged from 2 to 16 and either was comparable to or surpassed those of previously reported SSR markers. In previous studies conducted by Atallah et al. [41], Dunn et al. [42], Barari et al. [43], Mahalingam et al. [22], Mert et al. [44], Tok et al. [18], and Yu et al. [19], the maximum allele number did not exceed 10. In contrast, Aldrich et al. [45], Attanayake et al. [46], Buchwaldt et al. [23], Leyronas et al. [47], Peripolli et al. [48], and Sirjusingh and Kohn [10] reported ranges from 2 to 35 alleles. Additionally, the markers in the present study exhibited PIC values ranging from 0.1862 to 0.9014, with most classified as highly informative (PIC > 0.5). For comparison, previous studies reported PIC ranges as follows: Barari et al. [43]—0.388 to 0.781 for 3 markers; Sharma et al. [49]—0.03 to 0.806 for 25 markers; Peripolli et al. [48]—0.55 to 0.77 for 10 markers; Buchwaldt et al. [23]—0.126 to 0.949 for 47 markers. Notably, the marker SS23–1015 revealed 16 alleles and a PIC value of 0.9014, underscoring its high discriminatory power. These results indicate that the SSR panel developed here is not only comparable to but, in some respects, superior to previously published markers in terms of allele diversity and PIC. Such performance makes them sufficiently informative for population genetic studies and diagnostic assay development in *S. sclerotiorum*.

The marker development pipeline presented in this study demonstrates a robust potential for broader applications beyond *S. sclerotiorum*. The generated markers were short amplicon, experimentally validated, and evenly distributed across the genome, making them suitable for high-throughput genotyping, epidemiological surveillance, and molecular diagnostics in resource-limited contexts. Nevertheless, the present marker discovery phase utilized only three Korean genomes, which may not fully capture the global allelic diversity. Future validation across more geographically diverse and host-diverse isolates is essential to confirm the broader utility of these markers and refine their application in resistance breeding and pathogen monitoring.

Despite the largely clonal reproductive nature of *S. sclerotiorum* [50,51,52,53], each isolate in this study exhibited a unique SSR haplotype, reflecting high genotypic diversity, which is consistent with previous studies where all 127 Canadian isolates represented unique haplotypes [23]. Although *S. sclerotiorum* is known to be homothallic and self-fertile, replication slippage and point mutations, particularly within SSR-rich regions, are likely the primary sources of genomic variation [23]. Our UPGMA clustering analysis revealed no clear clustering by host or geographic origin, but this may reflect the limited number and regional concentrations of the Korean isolates. Previous studies on the association between SSR marker clusters and phenotypic traits in *S. sclerotiorum* have yielded mixed results. Some studies found no clear correlation between pathogenicity, geographic origin, or mycelial compatibility groups [18], whereas others reported isolate-specific associations between SSR alleles and partial resistance in sunflowers [14] or fungicide-induced variation in SSR loci [54]. In contrast, several other phytopathogenic fungi have shown more consistent links between SSR genotypes and phenotypic traits. For example, *Fusarium oxysporum* isolates clustered in alignment with their virulence patterns [55,56], *Alternaria alternata* groups showed fungicide sensitivity [57], and *Phytophthora capsici* SSR clusters contained metalaxyl-resistant genotypes [58]. In *Pyricularia oryzae*, SSR variation effectively tracked pathogenicity and host specificity [59].

Interestingly, the high genetic diversity in *S. sclerotiorum* has been documented at both micro- and macro-geographic scales in *S. sclerotiorum*. For instance, identical SSR haplotypes have been recovered from isolates collected more than 700 km apart [47], while distinct genotypes have been detected within a 1 m^2^ area [60]. Whether these patterns result from long-distance dispersal, localized selection, or host specialization remains unclear and warrants further investigation [45,61,62,63]. Although clear genotype–phenotype correlations remain elusive, the SSR markers developed in this study hold strong potential for future studies aimed at linking genetic variation to traits such as pathogenicity, fungicide resistance, and ecological adaptation.

In conclusion, the SSR markers developed in this study provide an efficient and transferable genotyping platform for *S. sclerotiorum*. Their high polymorphism, genome-wide coverage, and practical assay design support their applications with regard to epidemiology, population structure analysis, and pathogenicity/resistance screening.

## Figures and Tables

**Table 1:** Information on the *Sclerotinia sclerotiorum* isolates used in this study, including the host plant or substrate and the geographic origin.

Isolate No.	Year	Host or Substrate	Geographic Location
KACC 48152	2016	*Capsella bursa-pastoris*	Hwaseong-si, Gyeonggi-do
KACC 40923	2001	*Pimpinella brachycarpa*	Yeoncheon-gun, Gyeonggi-do
KACC 42223	-	*Chrysanthemum* sp.	Masan (Changwon)-si, Gyeongsangnam-do
KACC 45153	2010	*Brassica oleracea* var. *acephala*	Icheon-si, Gyeonggi-do
KACC 45771	2010	*Lactuca sativa*	Yuseong-gu, Daejeon
KACC 46833	2012	*Ranunculus asiaticus*	Hwaseong-si, Gyeonggi-do
KACC 46834	2012	*Anemone coronaria*	Icheon-si, Gyeonggi-do
KACC 47217	2013	*Ranunculus asiaticus*	Icheon-si, Gyeonggi-do
KACC 47218	2013	*Matthiola incana*	Suwon-si, Gyeonggi-do
KACC 47260	2013	*Matthiola incana*	Suwon-si, Gyeonggi-do
KACC 47261	2013	*Capsicum annuum*	Incheon, Gyeonggi-do
KACC 47709	2013	*Brassica oleracea* var. *italica*	Gangneung-si, Gangwon-do
KACC 47723	2014	*Allium tuberosum*	Iksan-si, Jeollabuk-do
KACC 47724	2014	*Foeniculum vulgare*	Iksan-si, Jeollabuk-do
KACC 47725	2014	*Malva sylvestris*	Iksan-si, Jeollabuk-do
KACC 47726	2014	*Matthiola incana*	Iksan-si, Jeollabuk-do
KACC 48153	2016	*Cucumis sativus*	Gongju-si, Chungcheongnam-do
KACC 48668	2018	*Codonopsis lanceolata*	Jeju-si, Jeju-do
KACC 48672	2017	Pod of *Pisum sativum*	Gangneung-si, Gangwon-do
KACC 48705	2018	Stem of *Canavalia ensiformis*	Jinju-si, Gyeongsangnam-do
KACC 40172	-	leaf of *Citrus sinensis*	Seogwipo-si, Jeju-do
KACC 40457	-	*Lactuca sativa*	unknown, Republic of Korea
KACC 40922	2001	*Pimpinella brachycarpa*	Namyangju-si, Gyeonggi-do
KACC 410245	2020	Stem of *Sedum sarmentosum*	Icheon-si, Gyeonggi-do
KACC 41064	1998	*Brassica napus*	Jeju-si, Jeju-do
KACC 41065	2000	*Capsicum annuum*	Hadong-gun, Gyeongsangnam-do
KACC 41069	1998	*Solanum tuberosum*	Jeju-si, Jeju-do
KACC 49781	-	Stem of *Brassica napus*	Yeongam-eup, Yeongam-gun, Jeollanam-do

**Table 2:** Whole-genome sequencing statistics data of *Sclerotinia sclerotiorum* isolates.

Isolate	Raw Data		Trimmed Data			Best Hit Reads		Covered Genome Length (bp)
	Reads	Bases	Reads	Bases	%	Reads	%	
KACC 42223	21,497,084	3,246,059,684	18,656,622	2,746,284,821	84.6	13,913,638	74.58	38,175,374
KACC 47260	22,409,198	3,383,788,898	21,352,534	3,184,738,647	94.12	18,293,424	85.67	38,249,628
KACC 410245	18,003,952	2,718,596,752	15,766,472	2,349,042,473	86.41	12,453,534	78.99	37,832,436

**Table 3:** Summary statistics of detected SNP and InDel variants of *Sclerotinia sclerotiorum* isolates.

Isolate	Total Variants	SNPs ^a^	InDels ^b^	Home Variants	Hetero Variants	Genic Variants	Intergenic Variants
KACC 42223	33,763	29,881	3882	28,073	5690	11,035	22,728
KACC 47260	31,336	27,740	3596	29,565	1771	10,874	20,462
KACC 410245	41,286	36,922	4364	36,925	4361	12,861	28,425

^a^ Number of single-nucleotide polymorphism (SNP) variants. ^b^ Number of InDel variants.

**Table 4:** Properties of selected polymorphic SSR markers, including motif, PIC, and allele number.

Marker	Chromosome	Position	SSR Motif	Primer (5′–3′)		TM (°C)	Product Size	NA ^a^	HE ^b^	PIC ^c^
SS14–1819	CP017814.1	1661050	(AGAGGAGGG)n	F:	GGGAGGGAATCTGAGATAAGTT	55.7	228	7	0.7013	0.6559
				R:	CAATAATTCCCTTTCCACTGAA	53.3				
SS15–1627	CP017815.1	2535712	(CATTCA)n	F:	CCTTACCTTACCTTACTTAACCTCC	57.1	237	8	0.8675	0.8339
				R:	GTGAGTTCGTTTCGTGTGTATG	57				
SS16–70	CP017816.1	1261826	(AC)n	F:	TCCGTACATACATCCATCCATA	55	172	3	0.2571	0.2338
				R:	ATTCTCAGCATACATTGTGTCG	55.9				
SS16–359	CP017816.1	2660538	(TAA)n	F:	AAACATACACTCAGCTGTTGAAA	55.9	231	14	0.8286	0.8030
				R:	AGGTCAGTGAAGGATGATTGTT	56.4				
SS17–256	CP017817.1	1606994	(TTG)n	F:	GAATAGCTATGCCTCCAGTGAC	57.4	219	12	0.8935	0.8658
				R:	CCAAATCCCAATCTTCTCATTA	52.9				
SS23–428	CP017823.1	799724	(ATGT)n	F:	GACTTGTCTCGAGCCTCTCTT	58.2	181	4	0.4182	0.3803
				R:	CCTCTGCACATCTTTAATTGGT	55.7				
SS23–1015	CP017823.1	538059	(TCAAATA)n	F:	CAAATGTATAAGTGCTGCCAAA	54.6	225	16	0.9247	0.9014
				R:	GGGAAAGGAAGACATTGATTTA	53.1				
SS26–345	CP017826.1	415037	(TTGC)n	F:	TTTGGATTTCTGATCTCGTACA	54.2	175	4	0.5429	0.4874
				R:	AGGAAGCTGCTCCTCTTCTC	57.8				
SS27–790	CP017827.1	1277078	(ATAGTT)n	F:	TGATATCGACAAGTGCTACGAG	56.8	230	5	0.7377	0.6786
				R:	TTCAGATGATGCAATAACCAAA	53.4				
SS28–205	CP017828.1	1019692	(GAT)n	F:	CGAGTATTATTGTGCAGATGGA	55.1	235	2	0.4675	0.3538
				R:	CCTCATTCAATACCTACCTACGTT	56.9				
SS28–258	CP017828.1	80138	(CATC)n	F:	GACCACGCCATATTGAACTAAT	55.7	239	2	0.4442	0.3411
				R:	GCTAGGTACTTGGTACACAGGC	59.4				
SS29–677	CP017829.1	153137	(AGCATCC)n	F:	GCTGTAGATAAACGGAGTCGTC	57.7	246	3	0.2000	0.1862
				R:	TAGAAATCGAGGACTATCCGG	55.4				

^a^ Number of alleles. ^b^ Unbiased expected heterozygosity. ^c^ Polymorphic information content.

## Data Availability

The raw genome sequencing data of Illumina NovaSeq X were submitted to the NCBI SRA database in a FASTQ format with BioSample SAMN48631575, SAMN48631576, and SAMN48631577 with SRA SRR33649454, SRR33649453, and SRR33649452 under BioProject PRJNA1011199.

## Supplementary Materials

The following supporting information can be downloaded at https://www.mdpi.com/article/10.3390/pathogens14070610/s1, Figure S1: A schematic workflow for the development and analysis of SSR markers in *Sclerotinia sclerotiorum*. The pipeline outlines the major steps including whole-genome sequencing data acquisition, variant calling, microsatellite (SSR) identification, primer design, and validation through PCR amplification and polymorphism analysis. Each stage integrates bioinformatic tools and filtering criteria to ensure the selection of robust and informative SSR loci for genetic characterization. Figure S2: Distribution of SSR motifs on each chromosome in *Sclerotinia sclerotiorum*.

## Abbreviations

The following abbreviations are used in this manuscript:

AD	Allele Depth
BAM	Binary Alignment/Map
bp	Base Pair
BWA-MEM	Burrows–Wheeler Aligner, Maximal Exact Match Algorithm
DP	Read Depth
ESTs	Expressed Sequence Tags
FS	Fisher Strand
GATK	Genome Analysis Toolkit
HE	Expected Heterozygosity
InDel	Insertion and Deletion
KACC	Korean Agricultural Culture Collection
MAF	Minor Allele Frequency
Mb	Megabase
MQ	Mapping Quality
MQRankSum	Mapping Quality Rank Sum Test
NA	Number of Alleles
PIC	Polymorphism Information Content
PCR	Polymerase Chain Reaction
QD	Quality by Depth
ReadPosRankSum	Read Position Rank Sum Test
SAM	Sequence Alignment/Map
SNP	Single-Nucleotide Polymorphism
SSR	Simple Sequence Repeat
UPGMA	Unweighted Pair Group Method with an Arithmetic Mean
VCF	Variant Call Format
