# Supplementary figures and images for: Development and Characterization of New SSR Markers in Sclerotinia sclerotiorum Using Genomic and Variant Analysis
Source: Pathogens. 2025 Jun 20;14(7):610. doi: 10.3390/pathogens14070610 (PMC12300245; doi:10.3390/pathogens14070610)

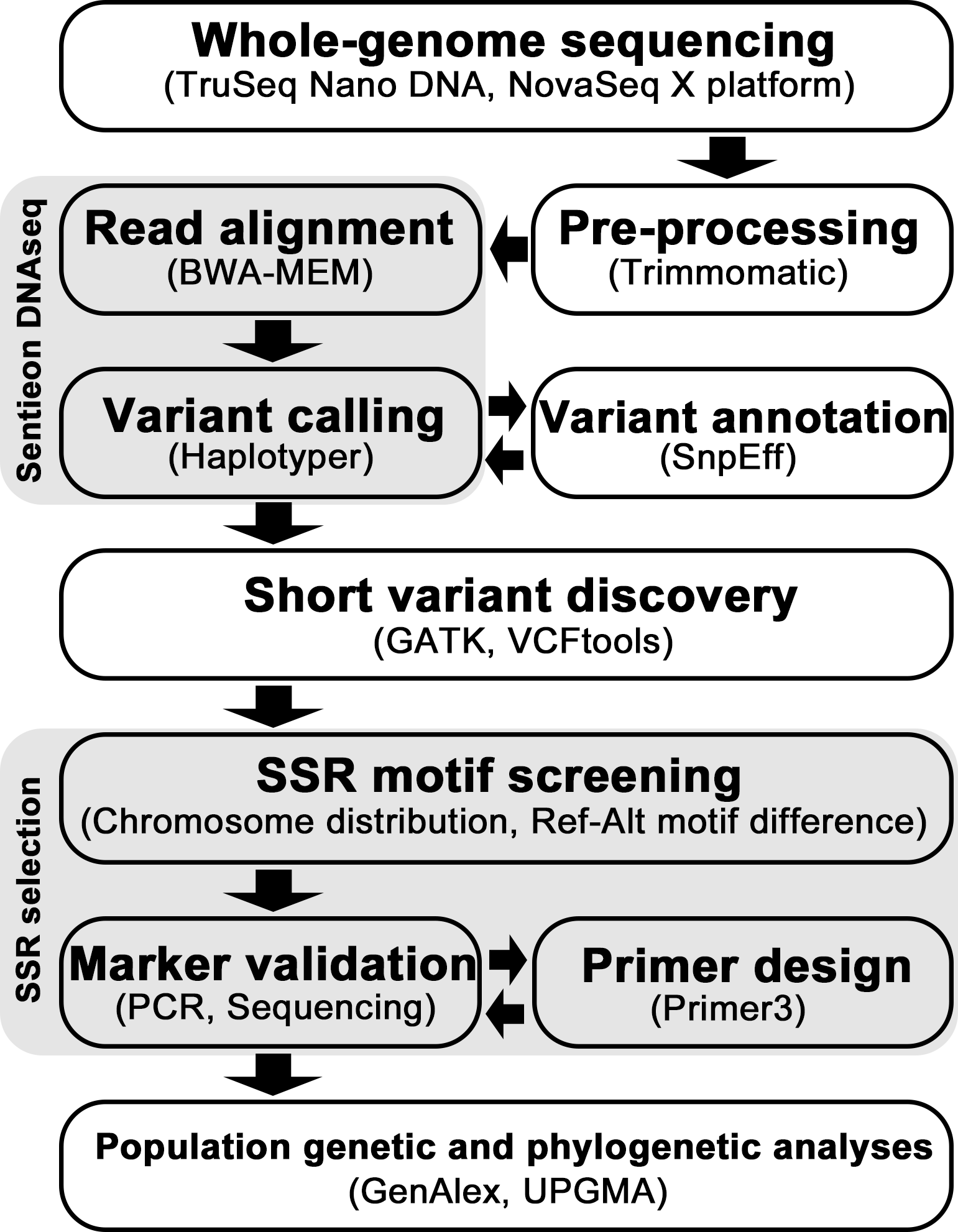

Supplement: Supplementary file 1 [file pathogens-14-00610-s001.zip › (SSR) Supplementary Figure S1_Schematic workflow for the development and analysis of SSR markers.tif]

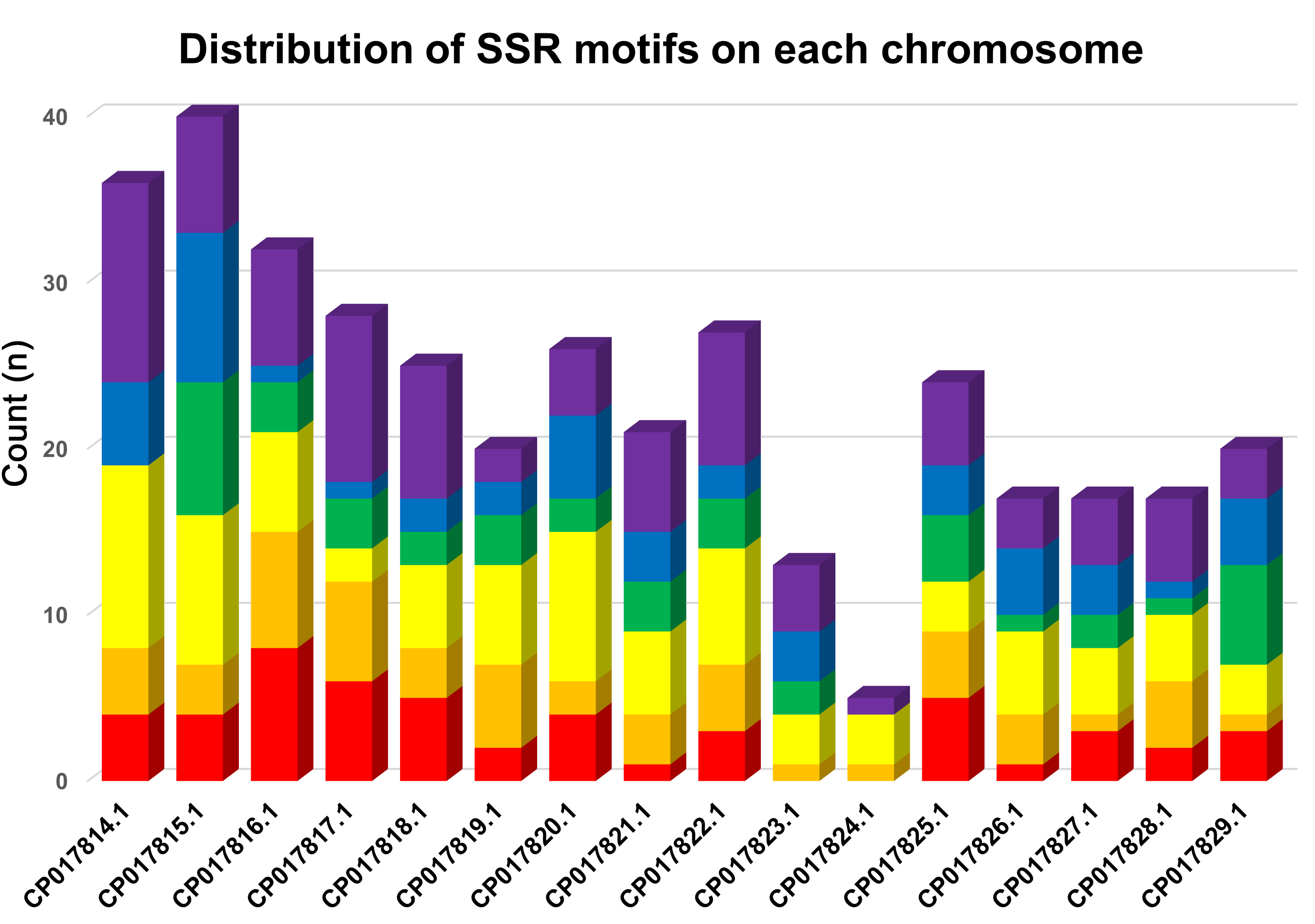

Supplement: Supplementary file 1 [file pathogens-14-00610-s001.zip › (SSR) Supplementary Figure S2_Distribution of SSR motifs on each chromosome in Sclerotinia sclerotiorum.tif]
